# An Anatomic Red Herring Found in the Diagnosis of Functional Vomiting

**DOI:** 10.7759/cureus.41978

**Published:** 2023-07-16

**Authors:** Varun Sharma, Aaron L Heston, Brian Lightwine, Ashish Patel

**Affiliations:** 1 College of Medicine, The University of Arizona College of Medicine - Phoenix, Phoenix, USA; 2 Pediatrics, Phoenix Children's Hospital, Phoenix, USA; 3 Radiology, Phoenix Children's Hospital, Phoenix, USA; 4 Pediatric Gastroenterology and Hepatology, Phoenix Children's Hospital, Phoenix, USA

**Keywords:** functional retching, non-billious emesis, congenital malformation, emesis, duodenum inversum

## Abstract

This report describes the case of a previously healthy 16-year-old patient who initially presented with emesis of unknown etiology that was refractory to standard medical interventions. The initial imaging revealed duodenum inversum, a rare anatomic abnormality that provided additional diagnostic complexity to this case. Though the final diagnosis was found to be functional vomiting, this case gives an instructive review of this rare anatomic abnormality, the significant effects it may cause, and how making a diagnosis of exclusion can be challenged by unusual turns in an otherwise straightforward presentation.

## Introduction

Duodenum inversum is a rare congenital malformation of the small intestine, with fewer than 20 cases reported in the literature [1-3]. In adult patients, it has been associated with other gastrointestinal (GI) malformations, including malrotation, annular pancreas, and pancreas divisum, though the finding is usually incidental and does not require surgical intervention unless there is evidence of obstruction [1]. Here, we present a case of functional vomiting, which is a diagnosis of exclusion [4], that was complicated by the radiographic finding of duodenum inversum.

## Case presentation

**Figure 1:**
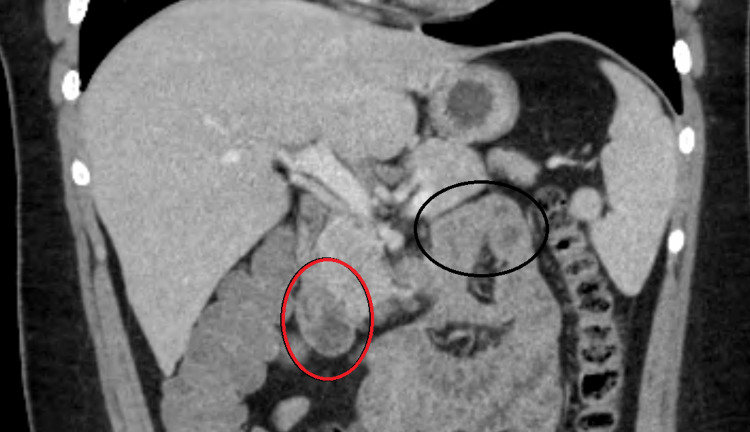
Abdominal CT scan (coronal) showing a nearly 180-degree directional relationship between the transition from the second to third segments of the duodenum (red oval) and the location of the duodenojejunal flexure (suspensory ligament, or ligament of Treitz) (black oval).

A previously healthy 16-year-old male with no significant medical or surgical history presented to the pediatric emergency department with a five-day history of non-bloody, non-bilious vomiting with correspondingly decreased oral intake. Throughout the course of illness, he had been afebrile without diarrhea, constipation, reflux, or other GI symptoms and he reported no morning or overnight emesis. He had initially presented to an adult hospital two days prior, where he reportedly received IV fluids and had normal blood work. A CT scan of his abdomen and pelvis (Figure 1) was obtained at this adult hospital without notable findings. He was sent home with scheduled Zofran but continued to have two to three episodes of emesis per day. A review by our pediatric radiologist upon admission also showed no concerning findings. However, after further workup and GI series with small bowel follow-through (described below), the team was able to identify directional components of the duodenum (marked in Figure 1).

Upon presentation to our institution, he appeared well and his vital signs were all within normal limits for his age. His admission BMI was 32 and he reported no recent changes in his weight or efforts to increase or lose weight. His initial complete blood count (CBC), comprehensive metabolic panel (CMP), lipase, and urinalysis (UA) were unremarkable, and his urine drug screen was negative. An abdominal x-ray showed no obstruction or significant stool burden. He vomited despite receiving Zofran, metoclopramide, and Benadryl, so he was admitted for further workup and supportive care.

**Figure 2:**
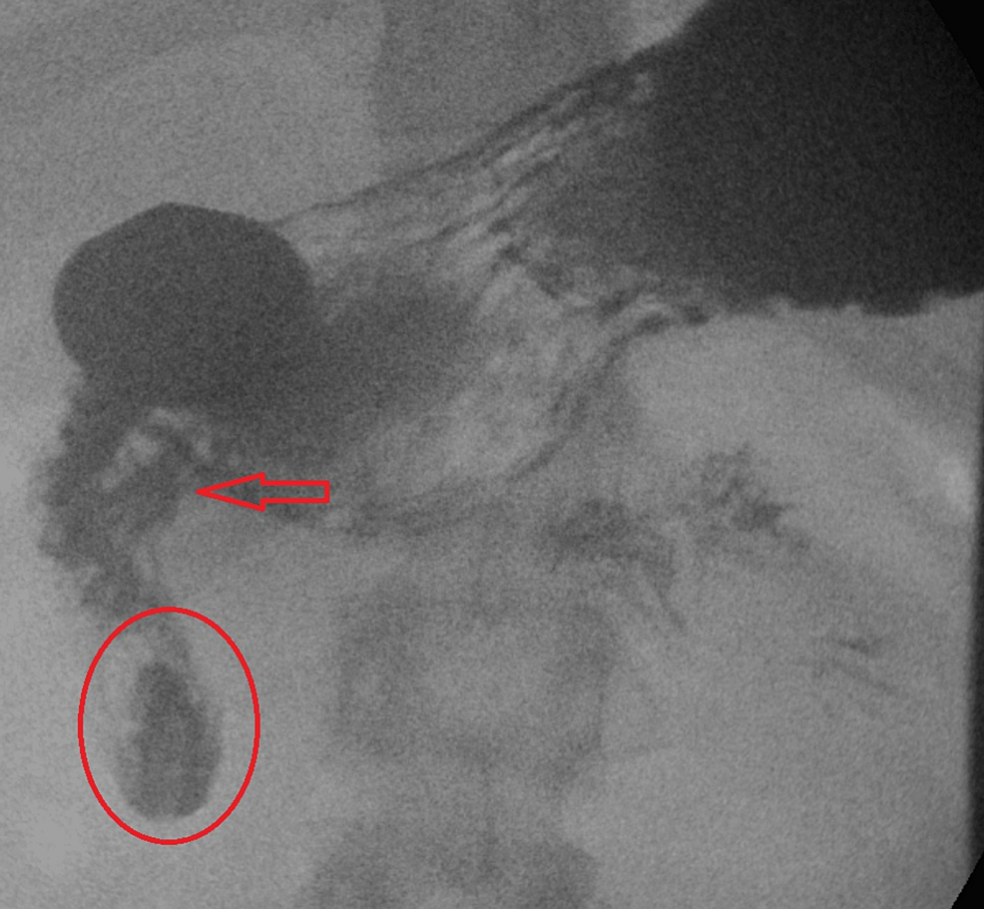
Small bowel follow-through showing path of duodenum demonstrating a 180-degree directional relationship between the transition from the second to third segments of the duodenum (red oval) and a lateral directional change of the third segment of the duodenum towards the left upper quadrant to the duodenal junction (red arrow).

**Figure 3:**
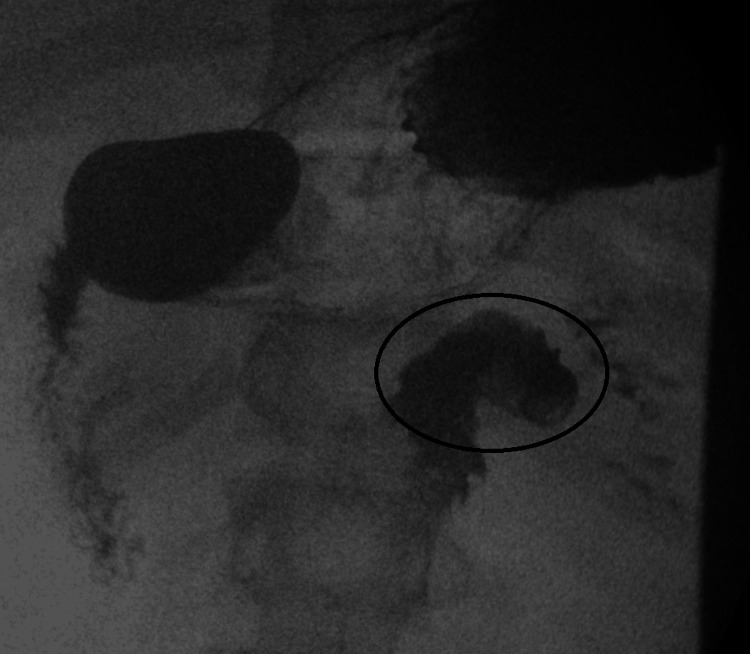
Small bowel follow-through showing duodenual flexure (black circle)

After admission, his oral intake remained minimal, and he continued to suffer from severe retching and emesis totaling nearly a liter of output daily despite scheduled antiemetics, proton pump inhibitor (PPI), and azithromycin. Three days after admission, with no clinical improvement, an upper GI series (UGI) study was obtained to reevaluate for intestinal obstruction (Figures 2, 3). This showed duodenum inversum but without the obstruction of contrast flow. Surgery was consulted who recommended no intervention due to the lack of obstruction. 

Gastroenterology was consulted and performed a diagnostic esophagogastroduodenoscopy, and all biopsies were negative for any pathologic diagnosis and had normal disaccharidase activity. Botox was also administered to the pylorus, which was tolerated without any adverse effects but provided no symptomatic improvement. Given his prolonged period of poor oral intake, a nasojejunal (NJ) tube was placed but quickly expelled due to the patient's vomiting. Because of this, the decision was made to use total parenteral nutrition (TPN) for nutritional and metabolic support. 

In combination with an otherwise normal workup, certain observations by the medical team brought suspicion for functional vomiting. It was noted that the patient would hold the emesis bag only during conversations with the providers, concurrently making loud retching sounds that were not productive of vomit. While engaged with preferred activities like playing videogames or watching television, these symptoms nearly disappeared, and he had no nighttime emesis while hospitalized. The psychology team was consulted and revealed a recent history of psychosocial stressors including struggles with academic performance and other family issues. When the patient was informed that the diagnosis was functional vomiting, he become visibly upset and immediately responded with further retching. 

He was discharged on amitriptyline and cyproheptadine, both of which help alleviate GI somatic sensation to ultimately aid in functional retching, and he continued with home TPN. He was closely monitored in the outpatient setting and demonstrated gradual improvement in vomiting symptoms and ability to tolerate oral intake. As his oral intake improved, his TPN was tapered and he was finally on a full oral diet six months after discharge.

## Discussion

The presentation of refractory vomiting initially offers a wide differential that must be sequentially evaluated before a functional cause of vomiting may be established. In the pediatric population, the most serious of these include intestinal malrotation, obstruction, superior mesenteric artery (SMA) syndrome, and self-induced vomiting [4]. Functional retching is classified as a disorder of gut-brain interaction. This is typically evaluated using the Rome criteria, which looks at the duration, frequency, and severity of symptoms. The incidence of these widely varies with patient age and gender. In this case, the finding of a rare anatomic abnormality added an additional layer of complexity to this diagnosis of exclusion.

In duodenum inversum, the third portion of the duodenum travels in a superior, posterior track before crossing the midline above the pancreas [1,3]. This differs from normal anatomy where the third portion travels horizontally across the midline prior to the fourth portion ascending to form the duodenojejunal flexure. The etiology of this is thought to result from the persistence of redundant dorsal mesentery within a mobile duodenum [5,6]. It is typically diagnosed through UGI. Though it is rarely described in the literature, its precise incidence is unknown. There are other case reports presenting it as an incidental finding obtained while investigating other comorbid GI conditions such as duodenitis and pancreatitis [2,5,7]. It is theorized that these conditions could be caused by stasis in the duodenal loop given the abnormal anatomic course. 

The most serious complication of duodenum inversum is GI obstruction, of which few pediatric cases have been described in the literature. One of these cases resulted in symptoms mimicking SMA syndrome which were ultimately resolved by partial Ladd’s procedure [8]. A more recent case presented with partial intestinal obstruction and was managed with medical management involving NJ feeds and acid suppression [9]. Two pediatric case reports attributed duodenum inversum with clinically significant gastroesophageal reflux disease (GERD), which was managed with both pharmacologic and surgical intervention [10]. The initial presentation of our case differed from these in both the acute time course without demonstrated obstruction and lack of unintentional weight loss.

## Conclusions

Making a diagnosis of exclusion is a laborious, stepwise process and requires a solid understanding of the wide differential. This makes it susceptible to distraction from medical red herrings as in this case. While duodenum inversum is a rare anatomical finding, its clinical significance is limited to its ability to produce acute GI obstruction or chronic GERD symptoms resulting in unintentional weight loss, neither were present in the history of presentation of this case.
